# Management of Isolated Radial Diaphyseal Fractures in the Paediatric Population

**DOI:** 10.7759/cureus.74467

**Published:** 2024-11-26

**Authors:** Shah Jehan, Helen Capitelli-McMahon, Jehan Zaib, Muhammad M Javaid, Rajesh Shah

**Affiliations:** 1 Trauma and Orthopaedics, York and Scarborough Teaching Hospitals, NHS Foundation Trust, Scarborough, GBR; 2 Plastic Surgery, Hull Royal Infirmary, Hull, GBR; 3 Trauma and Orthopaedics, Hull Royal Infirmary, Hull, GBR; 4 Trauma and Orthopaedics, Diana, Princess of Wales Hospital, Grimsby, GBR

**Keywords:** elastic nails, forearm fractures, paediatric fractures, radius fracture, ulna fracture

## Abstract

Introduction

Paediatric forearm fractures are common, but isolated radial diaphyseal fractures are rare, representing a small subset. Unlike fractures involving both the radius and ulna, these fractures lack well-established management guidelines. The potential for alignment loss during treatment underscores the need for specific protocols. This study highlights the importance of a tailored approach based on fracture classification. Stable fractures can be managed conservatively, but prompt surgical intervention is critical for unstable cases to prevent malalignment.

Methods

This retrospective study evaluated 597 paediatric forearm fractures surgically treated between 2011 and 2017. Of these, 49 cases of isolated radial diaphyseal fractures met the inclusion criteria. Patients with distal or proximal epiphyseal/metaphyseal fractures and those older than 18 years were excluded. To guide management, the study developed a simple classification system based on fracture pattern, angulation, and displacement.

Results

The fractures were classified into three groups based on a simple classification system developed for this study: stable fractures, moderately displaced fractures, and severely displaced or unstable fractures. Stable fractures, characterized by minimal angulation (<10°) and no significant displacement (<2 mm), were managed conservatively with immobilization. All 18 patients in this group achieved union without complications. Moderately displaced fractures, defined as angulation between 10° and 20° or displacement of 2-5 mm, typically required closed reduction, while five cases in this group underwent surgical fixation using elastic stable intramedullary nailing (ESIN). Outcomes for these patients were satisfactory, although some experienced mild complications such as transient stiffness. Severely displaced or unstable fractures, with angulation exceeding 20° or displacement greater than 5 mm, necessitated surgical intervention in all 15 cases. ESIN was the preferred method for stabilization, achieving good alignment and functional recovery, although one patient experienced transient nerve irritation. These results highlight the importance of a tailored approach to management based on the severity of fracture displacement and angulation.

Conclusion

The proposed classification and treatment protocol standardize management and improve outcomes for paediatric isolated radial diaphyseal fractures. Further research is required to validate these findings and refine treatment strategies for this rare injury.

## Introduction

Fractures of both the radius and ulna are common injuries in the paediatric population, accounting for 18%-25% of all paediatric fractures [1,2]. Falls are the predominant mechanism of injury, with boys more commonly affected than girls [2-5]. The most frequent forearm fracture is the distal metaphyseal-epiphyseal radius fracture, followed by fractures involving both the radius and ulna. In contrast, isolated fractures of either the radius or ulna, with or without radio-ulnar joint involvement, are relatively rare [6-8]. Specifically, isolated radial diaphyseal fractures constitute approximately 7.5% of all forearm fractures [6].

While management principles for isolated radial or ulnar fractures are well-established in adults, limited research has focused on isolated diaphyseal fractures in paediatric patients [9,10]. The classification of forearm fractures is typically based on the number of bones involved, fracture location, and radio-ulnar joint involvement [6]. However, a standardized classification for managing paediatric isolated radius fractures is notably absent, except for cases involving the proximal or distal radio-ulnar joint [11]. Recent studies suggest that isolated radial fractures may occur without involving the radio-ulnar joint [12,13].

Extensive research has been conducted on fractures of both forearm bones, leading to well-defined treatment protocols that consider factors such as age, fracture location, angulation, and displacement [14-16]. However, for isolated radial diaphyseal fractures, there are no clear treatment guidelines, and some studies have shown that these fractures are prone to losing alignment during treatment [6,17].

The purpose of this study was to retrospectively analyze the incidence, patterns of injury, and treatment options for isolated radial diaphyseal fractures in children. We also propose a simple classification system to guide management decisions at the time of presentation, aiming to support surgical teams in optimizing treatment outcomes. To our knowledge, there is no classification for isolated distal one-third radius fractures, which can help in the management of these fractures.

## Materials and methods

We identified 597 cases of paediatric forearm fractures requiring operative intervention at our institute between 2011 and 2017. The clinical notes and radiographs of these patients were reviewed to identify 49 cases with isolated radial diaphyseal fractures. Exclusion criteria included patients over 18 years of age, distal and proximal epiphyseal and metaphyseal fractures, open fractures, and those with associated ulnar shaft fractures.

The average age was 9.5 years (range: 3-16 years). There were 32 male and 17 female patients. The most common mechanism was falling onto an outstretched hand, followed by falling from a pushbike and falling playing football or rugby. The follow-up time ranged from six to 156 weeks, with an average follow-up time of 20 weeks. During routine follow-up, clinical and radiographic assessment was carried out at two weeks and then again at six weeks. At this point, patients were discharged if no metalwork was used and there was satisfactory healing. However, in cases with metalwork in situ or if there was any concern about healing, further follow-up was arranged. Most of the patients with metalwork had routine removal of metalwork after six months.

We classified the fractures based on two features: the site of the fracture and whether there was significant displacement or angulation associated with the fracture. Significant displacement was defined as at least 50% (half of bone diameter) translation on either the sagittal or frontal plane with any degree of angulation. Significant angulation was defined as more than 15 degrees of angulation in either the sagittal or frontal plane and less than 50% displacement. This was based on a previous study where authors used these criteria as indications for the reduction of paediatric forearm fractures [18].

**Figure 1:**
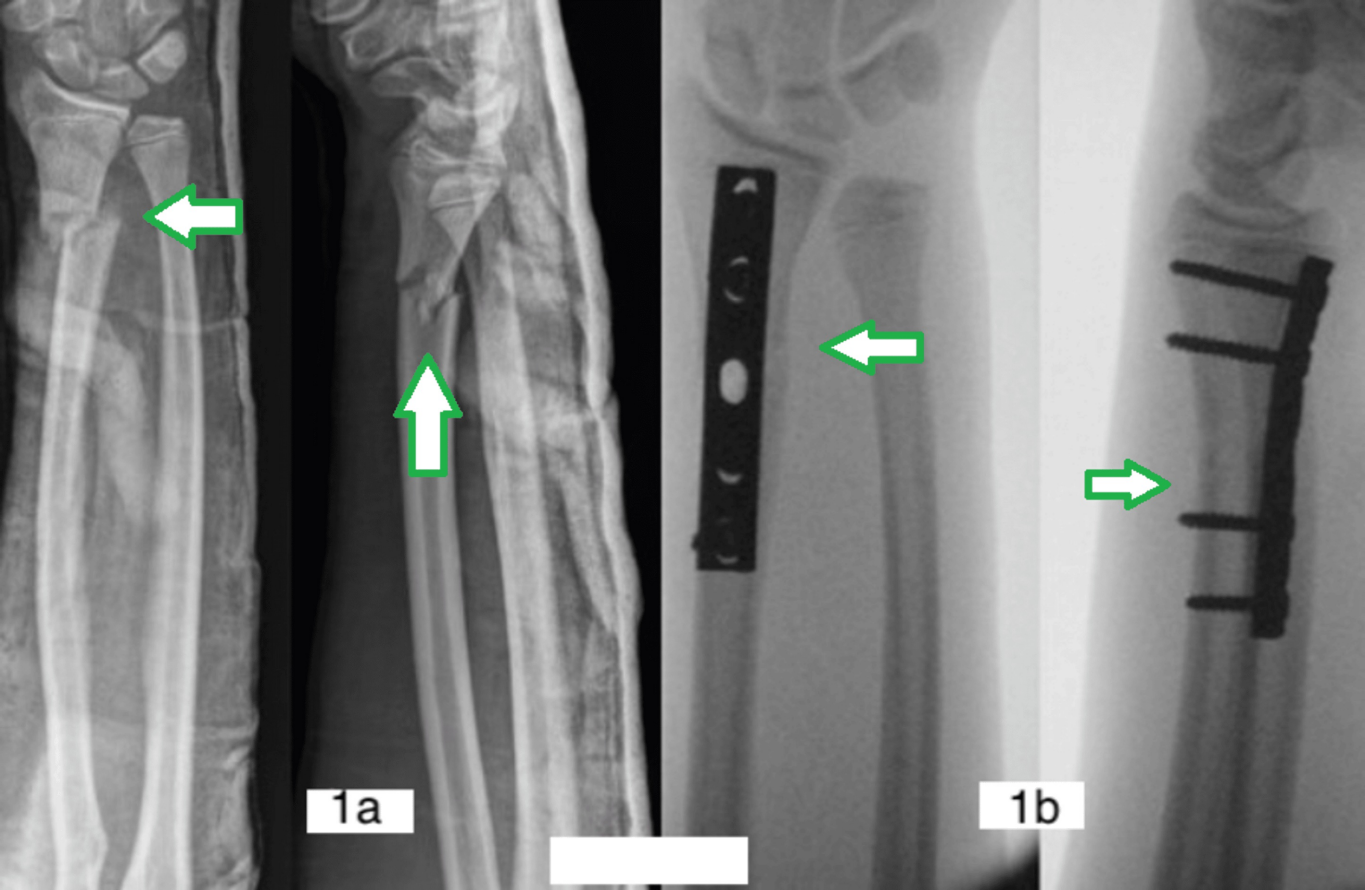
(1a) Displaced (arrow) distal one-third radius fracture; (1b) fracture reduction with plates and screws (arrow).

**Figure 2:**
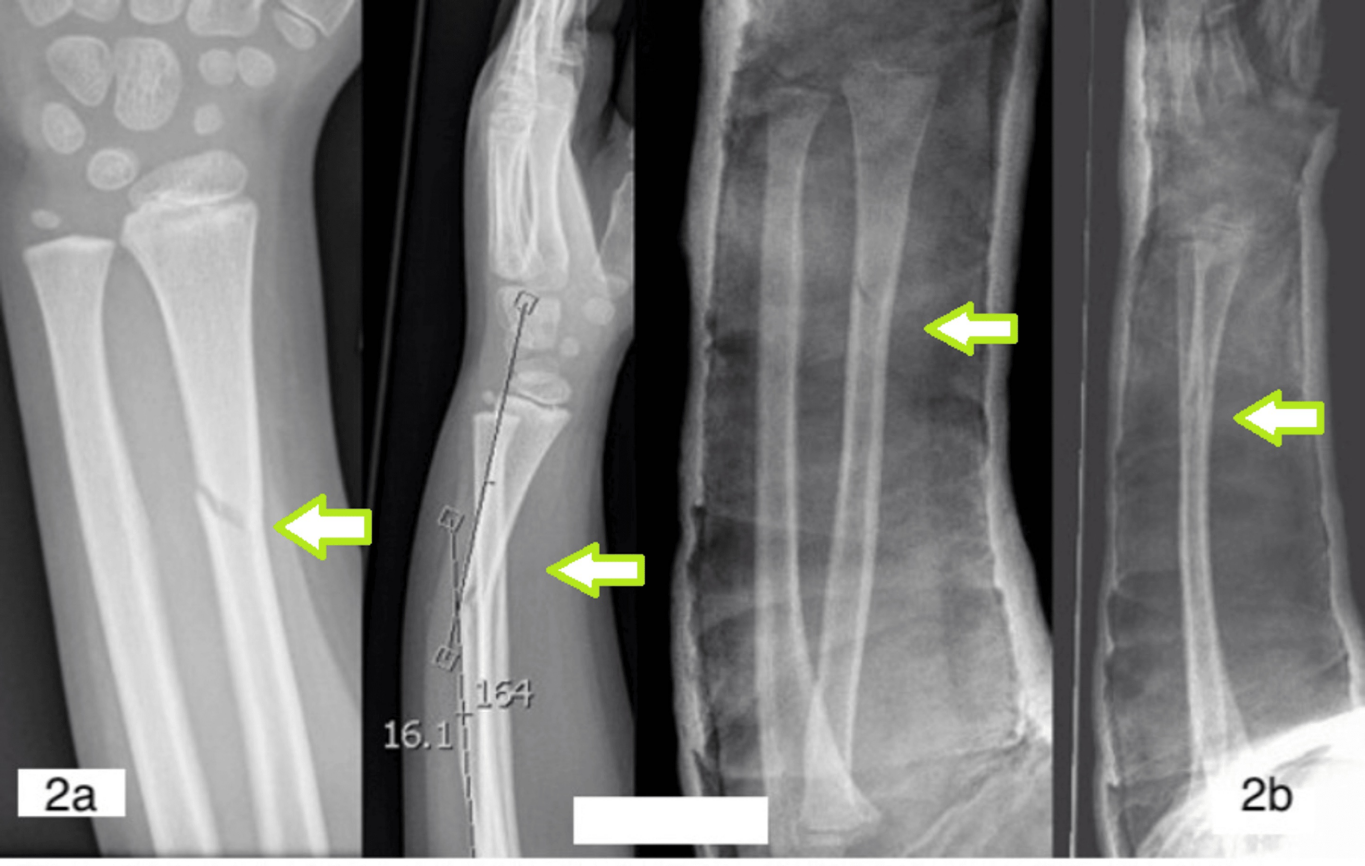
(2a) Isolated angulated distal one-third radius fracture (arrow); (2b) reduced fracture in plaster (arrow).

For defining a site, the radial diaphysis was divided into three equal zones on X-rays, giving us proximal, middle, and distal third fractures. The distal and proximal metaphyseal and epiphyseal fractures were excluded. Based on the site and displacement or angulation of the fracture, six patterns are possible. Each fracture type is given a two-part name. The first part indicates the site of fracture, i.e., distal, middle, and proximal third, and the second part indicates the type of fracture (displaced or angulated). We did not find any isolated displaced proximal third fractures in our series. The five fracture patterns identified in our series were distal third angulated (23), distal third displaced (13), middle third angulated (4), middle third displaced (5), and proximal third angulated (4) (Table 1). The typical radiographs for distal third displaced and distal third angulated fractures are shown in Figures 1, 2, respectively.

**Table 1:** Fracture types based on the fracture site, angulation/displacement, and treatment of each fracture pattern. * Further explanation is provided in the text regarding the indications of ORIF for these four patients. ORIF: open reduction and internal fixation; MUA: manipulation under anaesthesia; POP: plaster of Paris

Fracture Pattern	Total	ORIF With Plate	ORIF With Nail	MUA and With Nail	MUA and K-wires	MUA and POP
Distal 3rd angulated	23	4*	0	0	1	18
Distal 3rd displaced	13	11	0	1	1	0
Middle 3rd angulated	4	1	0	3	0	0
Middle 3rd displaced	5	0	4	0	0	1
Proximal 3rd angulated	4	0	0	4	0	0
Total	49	16	4	8	2	19

Patients' case notes and radiology records were reviewed to ascertain the management of each fracture type, complications, and outcomes. In particular, we looked at whether a satisfactory reduction was achieved with the closed reduction methods or whether it required open reduction. We also collected data for stabilization methods once satisfactory reduction was achieved. The stabilization methods with closed reduction methods included (1) manipulation under anaesthesia (MUA) and application of a cast, (2) MUA and flexible intra-medullary (IM) nails, and (3) MUA and K-wiring. Wherever open reduction was required, the stabilization was achieved with the use of a plate or a flexible IM nail.

We correlated the management with different fracture patterns. The difficulties faced with closed reduction for some fracture patterns were recorded. The complications and subsequent re-operations were recorded and correlated with the initial fracture pattern and initial treatment.

## Results

Table 2 shows data for the five fracture patterns and the treatment of each fracture type. We found 36 isolated fractures of the distal third of the radial diaphysis. These included 23 fractures with significant angulation and 13 with significant displacement. The average angulation was 23° (range, 15-38°). In the angulated group, 18 patients were treated with MUA and an above-elbow cast, one had MUA with K-wire insertion, and four had an open reduction and internal fixation with plating. Of the four patients who underwent open reduction and plating, one was a mature adolescent patient, and, therefore, the decision was made to do an open reduction and internal fixation with a plate. The second patient was a six-year-old child whose angulated fracture was initially treated in a cast as the position was acceptable. However, subsequent X-rays showed increased angulation with callus formation. Therefore, the decision was made for open reduction and internal fixation with a plate. The third child presented late, at 18 days after the initial injury, with the angulated fracture in an unacceptable position, while the fourth patient presented as a re-fracture through a previously angulated distal third fracture.

**Table 2:** Complications and their treatment. MUA: manipulation under anaesthesia; ORIF: open reduction internal fixation; DRUJ: distal radio-ulnar joint

Complication	Age	Fracture Type	Initial Management	Management of Complication
Loss of fracture position and DRUJ subluxation	14	Distal third angulated	MUA and cast	MUA and Nancy nail Radius + ulna osteotomy and fixation with plate (Figure 3)
Dorsal angulation deformity	9	Distal third angulated	ORIF with plate	Removal of metal work 3 years after the first surgery with no further intervention (Figure 4)
Delayed wound healing	11	Distal third displaced	ORIF with plate	Regular dressings, no surgery needed
Pin site infection	6	Distal third displaced	MUA and K-wires	Oral antibiotics
Periprosthetic fracture and then dorsal angulation	6	Distal third displaced	ORIF with plate	Removal of plate and fixation with a new plate, then removal of the plate

In the group with displaced fractures, 11 out of 13 patients were treated with open reduction and internal fixation; these were all older children with an age range of 10-16 years. In four of these 11 cases, an initial attempt was made to perform a closed reduction and stabilization with a flexible IM nail. However, this was unsuccessful, and therefore, open reduction and stabilization with a plate were required. With a displaced isolated radius fracture with 100% translation, we think close reduction is very difficult due to periosteal interposition. Another factor is an intact ulna, which prevents reduction by longitudinal traction. Two patients with displaced distal third fractures underwent closed reduction. The first child was a four-year-old who was treated with a flexible IM nail, and the second child was a six-year-old who was treated with MUA and K-wires. In both of these patients, the translation was less than 100%.

We had nine cases of middle third fracture, four of which were angulated and five of which were displaced. Of the four angulated fractures, three were treated with closed IM nailing. One case was initially treated with an above-elbow cast. However, three weeks later, radiographs showed increased angulation, and therefore, open reduction and plating were required.

In the displaced middle third group, four patients were treated with open reduction and IM flexible nails. The fifth patient, a four-year-old, underwent MUA with an above-elbow cast. Although the fracture reduction was suboptimal, it was accepted in view of the patient's young age. At the last follow-up, the fracture had healed satisfactorily.

We had four cases of proximal third fractures, which were all angulated fractures. We did not identify any displaced isolated proximal third radial diaphysis fractures in our cohort. Of these four, one patient was more complex, as the fracture sustained was a re-fracture of the proximal radius from a previous fracture of both bones. The fracture angulation, in this case, could not be improved with manipulation alone. Of the remaining three fractures, one fracture was found to be unstable on closed reduction and, therefore, underwent closed IM nailing. The remaining two were initially treated with an above-elbow cast, as the initial angulation was minimal. However, at one-week follow-up, deterioration in the anterior angulation was noted. Hence, both underwent closed reduction and flexible IM nailing.

Complications occurred in five cases; all five were fractures of the distal third. These included malunion with dorsal angulation (1), loss of position with dislocation of the distal radio-ulnar joint (1), delayed wound healing (1), pin site infection (1), and a fracture distal to a plate (1). The complications and their management are shown in Table 2.

**Figure 3:**
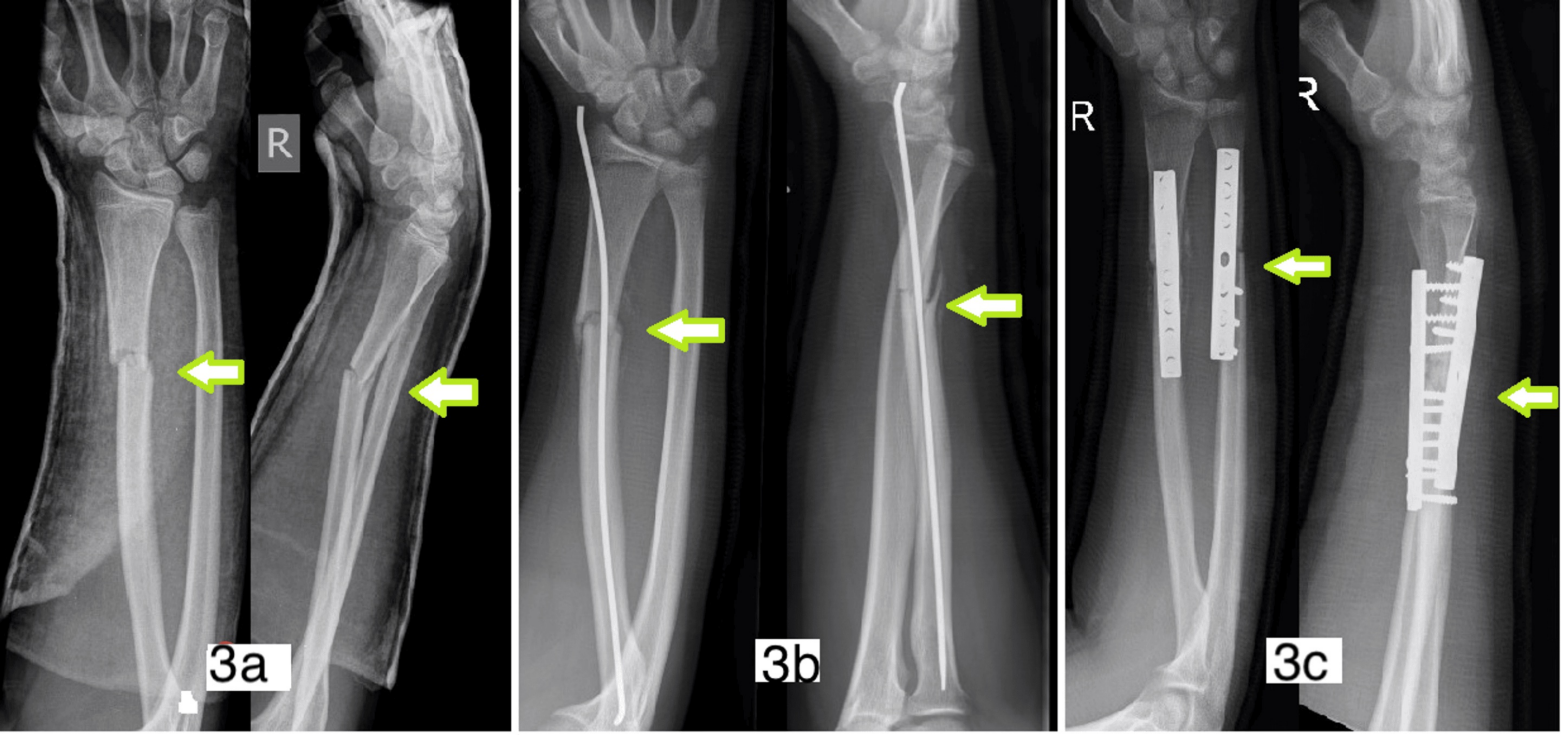
(3a, 3b) Volar angulation and disruption of the radio-ulnar joint after elastic nailing; (3c) open reduction and plate fixation of the same fracture.

**Figure 4:**
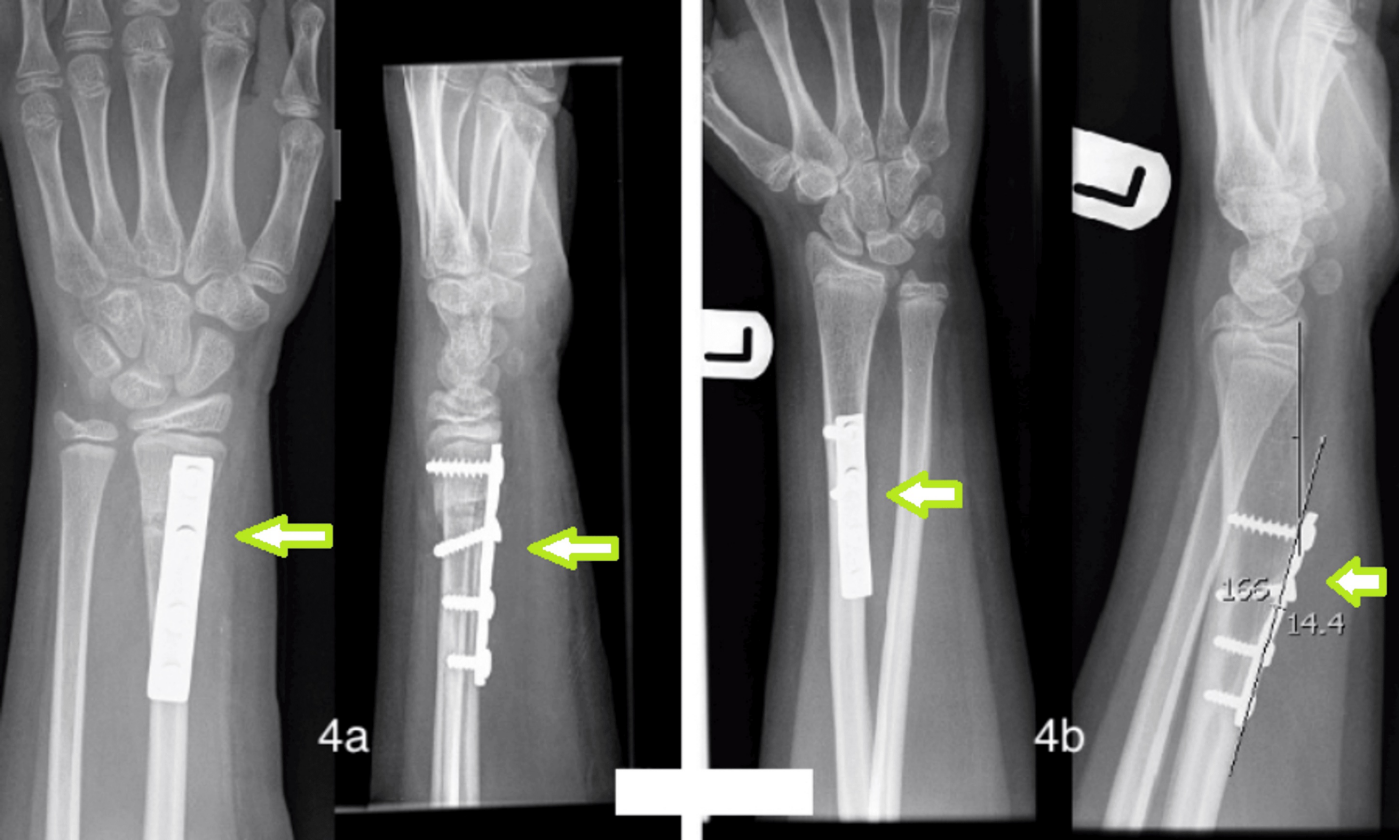
Plate fixation of the angulated distal one-third radius fracture.

The first patient was a 14-year-old boy who had a distal third angulated fracture. He initially underwent closed reduction and an above-elbow cast. At the one-week follow-up, radiographs showed a loss of position. At this point, the patient underwent closed reduction and flexible IM nailing. Despite this, there was volar angulation of the radial shaft fracture at four weeks and disruption of the distal radio-ulnar joint (Figure 3). This then required an osteotomy of both the radius and ulna with plating of both bones to correct the alignment and restore the distal radio-ulnar joint. The second patient had open reduction internal fixation for refracture of the distal radius. Even with satisfactory reduction, the patient developed dorsal angulation deformity (Figure 4). The metalwork was eventually removed with no further intervention.

The third patient had open reduction and internal fixation of the displaced distal third fracture. He had mild dehiscence of the superior part of the wound. His wound eventually healed without any intervention.

The fourth patient was a six-year-old boy who underwent K-wiring and developed a K-wire site infection. This was treated with antibiotics and required no further operative treatment.

The fifth patient, a six-year-old boy, had a fracture just distal to the plate following a fall four months after the initial (uncomplicated) surgery. He underwent revision with a plate. The same patient then developed dorsal angulation deformity. The metalwork was eventually removed. Unfortunately, he was lost to follow-up after the removal of the metalwork. His deformity would likely have been remodelled as he was only nine years old at the time of the metalwork removal.

## Discussion

This study reviewed the management of isolated radial diaphyseal fractures in children. There is a paucity of published literature on the incidence and management of these fractures. These isolated radial shaft fractures are infrequent but not rare fractures in the paediatric age group [13]. Perhaps this infrequency is one of the reasons that this fracture is not as well addressed in the literature as it is for adults. Grabala [6], in his study of 1,668 paediatric forearm fractures, found an incidence of 7.55% (126/1,668) of isolated diaphyseal fractures of the radius. However, he did not elaborate further on the level of these fractures. Alrashedan et al. [19], in their study of 318 paediatric forearm fractures, found that isolated radial shaft fractures accounted for 9.43% of their cases, with the distal third accounting for 6.6% and the middle third accounting for 2.83%. Their series did not have any isolated proximal third radius fractures. These two studies were epidemiological studies and did not provide further information on the treatment modalities for these fractures. Our study showed an incidence of 8.2% (49/597) of isolated radial shaft fractures, which is consistent with the above studies.

Guitton et al. [20] found that 10 of 13 (76.9%) patients with isolated radial diaphyseal fractures were treated satisfactorily with manipulation and an above-elbow cast, while the other three patients had plating, but it would appear that the decision to operate was based on the surgeon's preference. Further information regarding the level and displacement for fractures was unavailable. In their series, Walsh et al. [21] found that 36 of 41 patients (87.8%) with Galeazzi fracture dislocation in children had excellent or fair results after treatment with manipulation and cast. However, their study did not elaborate on the degree of displacement or the angulation of the fractures sustained by their cohort. The patients in their study were treated in a variety of ways, including below-elbow casts, above-elbow casts in varying positions, and open reduction. Eberl et al. [22], in their study of 26 Galeazzi fractures in children, noted that 22 patients (84%) were treated with manipulation and casting. Nine patients were immobilized in an above-elbow cast and the other 13 in a below-elbow cast, with the results being better in the children treated with a below-elbow cast. Again, there was no information about the displacement or the angulation of the fractures that were treated.

Overall, the review of the current literature does not provide a very clear strategy for the management of these fractures, and the information available is especially scarce on the management of isolated middle and upper third fractures. We classified these fractures based on angulation, displacement, and fracture site to look for patterns and suggest management based on fracture patterns.

In our series, 18 of 23 (78%) of the angulated fractures of the distal third radial shaft were treated successfully with manipulation and an above-elbow cast. This is consistent with the results noted above [19,21]. Despite the findings of Eberl et al. [22], we would agree with Walsh et al. [21] and suggest an above-elbow cast.

However, in the displaced distal third radial diaphyseal fractures, we found that 11 of 13 (85%) required open reduction and internal fixation with a plate. It is important to note that attempts were made initially to reduce these fractures closed, but this was not possible. In our experience, if there is 100% translation, then reduction by closed methods becomes very difficult. This is probably due to the intact ulna preventing correction with longitudinal traction and also possibly due to soft tissue interposition at the fracture site. This adds weight to the conclusion that for these fractures, it may be prudent to proceed straight to open reduction and internal fixation with a plate. This is at variance with the above studies [22,23]; however, this may be due to the lack of clarity in the above studies regarding the displacement of the fractures.

Displaced distal third radius shaft fractures are often associated with disruption of the distal radio-ulnar joint, and restoration of the radial length remains important. In Walsh et al.'s [21] study, the majority of the fractures were in the age range of 9-12 years, while in our cohort, for those requiring open reduction and stabilization with a plate, the age range was from 10 to 16 years. The remaining two patients who did not require open reduction were aged four and six years. As noted above, we had a complication when a flexible nail was used to stabilize the distal third angulated fracture in a 14-year-old. The procedure failed to prevent further angulation of the fracture and disruption of the distal radio-ulnar joint (Figure 3). This subsequently required plating with an osteotomy to correct the persistent deformity. Based on our findings, we would suggest that displaced isolated fractures of the distal third radial diaphysis in the adolescent age group have a high probability of requiring open reduction, and we would recommend internal fixation primarily with a plate. These fractures are different from the common metaphyseal fractures, which obviously can be treated with manipulation and cast or manipulation and stabilization with K-wires.

Eberl et al. [22], in their study of Galeazzi fractures in children and adolescents, found that only eight of 26 (31%) cases were correctly diagnosed as having a Galeazzi fracture at the time of their initial treatment. Walsh et al. [21] found in their retrospective study that in 17 of 41 (41%) cases of Galeazzi fracture, dislocation of the distal radio-ulnar joint was not recognized at the time of the initial presentation. They recognized the difficulty of obtaining appropriate radiographs in the paediatric population. To avoid confusion, we have not used the terms 'Galeazzi' and 'Galeazzi variant' as we feel that classifying the fracture into angulated and displaced is more straightforward and easier to determine on the initial radiographs. It must be stressed that in all cases, satisfactory reduction of the distal radio-ulnar joint must be achieved intraoperatively and then maintained.

In our series, three out of four (75%) angulated middle third fractures were treated with closed manipulation and an above-elbow cast. For the displaced fractures, again, four out of five (80%) cases required open reduction to achieve a satisfactory reduction. In this subgroup, stabilization with a flexible IM nail proved satisfactory. The fifth patient was a four-year-old, for whom, in view of his age, a suboptimal position following a closed reduction was accepted. Therefore, it would be reasonable to suggest that displaced middle third isolated radial diaphyseal fractures will likely require open reduction. As stated above, this is probably due to the intact ulna preventing correction with longitudinal traction and possibly soft tissue interposition at the fracture site.

In our series of 597 cases, we were unable to identify any displaced isolated proximal third radius fractures. There were four angulated fractures of the proximal radius. All of these were stabilized with flexible IM nailing. Based on our experience, we suggest that closed flexible IM nailing should be considered for angulated proximal third fractures. If a decision is made to treat this fracture in an above-elbow cast without stabilization with a flexible IM nail, then we suggest a very close follow-up with a radiographic assessment. Our experience indicates that deterioration in angulation within the cast is likely. This is evidenced by the displacement that occurred in two cases in our series that were initially treated only in a cast. The deforming force is likely biceps insertion into the proximal fragment, which results in apex anterior angulation.

It is not surprising, considering the nature of the injury, that our numbers for the middle third and upper third fractures are not high, but the overall pattern of the injury and its treatment has remained consistent. We were unable to accurately determine the dexterity, mechanism, and environment of injury to assess if this contributed to the subsequent treatment required. However, the study focused on identifying the patterns of isolated radius diaphyseal fractures and an improved system for classifying these fractures and their management.

Other limitations of our study include data being collected retrospectively. Therefore, details on outcomes focusing on a range of movement and dexterity in each group are limited. Similarly, some patients were lost to follow-up, which limited our dataset. We defined 'acceptable' or 'not acceptable' degrees of angulation or displacement based on a previous study by Colaris et al. [18]. Their work was based on both-bone fractures, while our study assessed isolated radial shaft fractures. We are hopeful that, in view of the paucity of literature on the management of this important subgroup of forearm fractures, our study will prove helpful in determining the most appropriate management.

## Conclusions

This is probably the most extensive study on the management of isolated radial diaphyseal fractures in the paediatric age group in the published English literature. Our analysis concludes that the majority of angulated isolated paediatric radial diaphyseal fractures can be reduced using closed methods, whereas displaced isolated radial fractures more often require open reduction. In this series, the majority of the distal third displaced fractures were fixed with a plate, and the middle third displaced fractures were treated with open reduction and flexible IM nails. Our study provides a detailed classification of isolated paediatric radial diaphyseal fractures and their management; however, further validation is required.
